# Impact of the COVID-19 Pandemic on the Lifestyles and Quality of Life of Women With Fertility Problems: A Cross-Sectional Study

**DOI:** 10.3389/fpubh.2021.686115

**Published:** 2021-07-19

**Authors:** Gemma Biviá-Roig, Ana Boldó-Roda, Ruth Blasco-Sanz, Lola Serrano-Raya, Elena DelaFuente-Díez, Pedro Múzquiz-Barberá, Juan Francisco Lisón

**Affiliations:** ^1^Department of Nursing and Physiotherapy, Faculty of Health Sciences, University CEU-Cardenal Herrera, CEU Universities, Valencia, Spain; ^2^Department of Gynecology and Obstetricia, La Plana University Hospital, Vila-Real, Spain; ^3^Department of Gynecology and Obstetricia, Hospital of Sagunto, Valencia, Spain; ^4^Department of Biomedical Sciences, Faculty of Health Sciences, University CEU-Cardenal Herrera, CEU Universities, Valencia, Spain; ^5^CIBER of Physiopathology of Obesity and Nutrition CIBERobn, Carlos III Health Institute, Madrid, Spain

**Keywords:** female infertility, COVID-19, confinement, lifestyle, quality of life, diet, anxiety, depression

## Abstract

**Background:** The COVID-19 pandemic has implied worldwide the imposition of confinement measures and mobility restrictions, to a greater or lesser extent. It has also meant the closure of some public medical services such as reproductive care. This situation may have impacted the health-related behaviour and quality of life of women with fertility problems.

**Objective:** The objective of this study was to analyse the effects of confinement and the suspension of reproductive medical care on the lifestyle (diet, physical exercise, and smoking habits), anxiety and depression, and quality of life of infertile women by comparing their pre- and post-confinement situations.

**Methods:** We carried out a cross-sectional, internet-based study. Information was collected on these women's adherence to the Mediterranean diet (MEDAS questionnaire), physical exercise (IPAQ-SF), anxiety and depression (HADS), and quality of life related to fertility (FertiQol) before, during, and after confinement. The survey was conducted between 1 September and 28 October 2020.

**Results:** A total of 85 women participated. There had been a significant increase in anxiety and depression levels (*P* < 0.001) and an increase in tobacco consumption among female smokers during confinement vs. pre-confinement (62.5% had increased their consumption). The participants had also increased the mean number of hours they spent sitting (*P* < 0.001). There had also been an increase in vigorous and moderate exercise levels by 40 and 30%, respectively (*P* = 0.004). However, no differences were observed in these patients' eating habits as a result of confinement (*P* = 0.416). When the reproduction service was resumed, the participants showed higher anxiety level scores (*P* = 0.001) with respect to the pre-confinement situation as well as lower mean FertiQol scale score (*P* = 0.008).

**Conclusions:** Confinement had increased anxiety and depression levels among these infertile women as well as tobacco use among the participants who were smokers. The prolonged closure of reproductive care units decreased the quality of life of the participants of this study. These results suggest the need to implement online programs to improve healthy habits and quality of life of this population group.

## Introduction

The COVID-19 disease caused by the SARS-CoV-2 virus first appeared in China at the end of 2019. Its rapid spread, together with the severity of the disease, generated an alarming increase in the number of hospital admissions to wards and intensive care units. Faced with these high patient burdens, hospitals in many of the most affected countries, including Spain, were forced to redistribute their medical resources and health personnel to care for patients who were sick with COVID-19. This health emergency led to the suspension of medical procedures and non-urgent surgical interventions (1). In the field of reproductive medicine, the American Society for Reproductive Medicine and the European Society of Human Reproduction and Embryology recommended indefinite interruption of reproductive care during the early stages of the pandemic (2).

Difficulty in conceiving is a major source of stress, anxiety, frustration, and even depression (3–5) and can result in a decrease in the quality of life of couples, but especially among women (6). Many women have had to deal with fertility problems during this unusual situation of the pandemic, which has been marked by the suspension of reproductive care and implementation of social distancing and home confinement. The strict confinement rules adopted by several countries implied a drastic modification in the daily routines of the population. However, in the case of women of childbearing age, lifestyle plays a fundamental role in their reproductive health.

In addition, many women trying to conceive lead unhealthy lifestyles that affect their chances of becoming pregnant (7, 8). Risk factors include poor diet (9, 10), being overweight or obese, a sedentary lifestyle (11), tobacco use, and high levels of anxiety or depression (12), among others (13). Moreover, women's pre-conception lifestyle can not only decrease natural fertility, but also affect the results of fertility treatments. Thus, various studies have shown that factors such as a high body mass index (14–16) or an unhealthy diet (10, 17–19) have a negative impact on implantation and pregnancy rates in women undergoing re-productive treatments. Therefore, the clinical guidelines on fertility recommend that women planning pregnancy should maintain a healthy and balanced diet, regularly en-gage in physical exercise, and stop smoking in the case of those who smoke (20, 21).

Before attending reproduction consultations, many women will have been trying to conceive for several months or years (22). The current pandemic scenario has forced the postponement of reproductive health care appointments for many women, which can entail potential added stress. In fact, the results of a recent survey of patients undergoing fertility treatment shows that 85% were moderately to extremely distressed by the treatment cancellations because of COVID-19 (23). In this context, the objective of this cross-sectional study was to analyse the impact of confinement and the closure of reproduction services as a result of COVID-19 on the lifestyle (diet, physical exercise, and smoking habits), anxiety and depression, and quality life of women with fertility problems who had been referred to reproduction services, by comparing their pre- and post-confinement situations.

## Materials and Methods

This was an internet-based, cross-sectional survey. The questionnaire used in this work was disseminated to women with fertility problems by the Reproduction Service at a public hospital located in the Valencian Community (Spain). To access the survey, the participants were provided with a link created using the Google Forms tool which allows the creation of online surveys and automatically collects the resulting data in a spreadsheet. The survey was conducted according to the Checklist for Reporting Results of Internet E-Surveys (24). The data were collected from 1 September 2020 (when the hospital's reproduction service was reopened) until 28 October 2020 (when a state of alarm was declared again in Spain in conjunction with new mobility restrictions). Each participant responded to the survey only once. The questionnaire collected information about three moments during the pandemic: before confinement, during confinement, and when the reproduction service reopened.

Infertile women aged between 18 and 38 years who had been referred to the Hospital's Reproduction Unit for primary infertility and who were waiting for an appointment when home confinement was imposed in Spain (15 March 2020) were included in this study. Non-Spanish speaking women with difficulties understanding Spanish language or who had a diagnosis of a psychiatric disorder were excluded.

The online questionnaire comprised 60 items which collected the following information:

- Sociodemographic characteristics (age, height, weight, educational level, time spent trying to get pregnant, and employment status).
- Eating habits were assessed using the Mediterranean Diet Adherence Screener (MEDAS) questionnaire from the PREDIMED study. This questionnaire, which has been validated for the Spanish population, assesses adherence to the Mediterranean diet. It consists of 14 items, of which 12 assess the frequency of food consumption, and the other two examine adherence to the characteristic dietary habits of the Spanish Mediterranean diet. Each item is scored from 0 or 1, and based on the total score, the participants are classified as having a low-adherence (score of 0–5), medium-adherence (score of 6–9), or high-adherence (score ≥ 10) to the Spanish Mediterranean diet (25).
- Physical activity levels were examined using the abbreviated version of the International Physical Activity Questionnaire (IPAQ short-form), which has been vali-dated for the Spanish population (26). This questionnaire collects specific information about the days per week and minutes per day these women spent engaged in vigorous or moderate exercise, walking, or sedentary activities. In addition, general information was collected on their perception of this exercise and perceived obstacles to engaging in exercise during confinement.
- Smoking habits were recorded as the number of cigarettes smoked per day.
- Anxiety and depression were assessed using the Hospital Anxiety and De-pression Scale (HADS) (27) which comprises 14 questions, of which 7 assess anxiety symptoms (HADS-Anxiety) and 7 measure symptoms of depression (HADS-Depression). Each item is scored from 0 to 3, with a score range on each sub-scale of 0–21 points. Scores of 0–7 indicate the absence of anxiety or depression; scores of 8–10 indicate mild levels; scores of 11–14 indicate moderate levels; and scores of 15–21 indicate severe levels. This scale has been validated and had adequate psychometric properties in infertile patients (28).
- The quality of life related to fertility problems was explored using the Fertility quality of life tool (FertiQol) (29), which comprises 24 items that assess the impact of the emotional, mind-body, relational, and social domains related to fertility problems. Each item is assigned a value between 0–4 and higher scores on the scale indicate better quality of life in relation to fertility. This questionnaire has been previously validated in 6 countries and translated into multiple languages, including Spanish, and shows adequate psychometric properties (30).

### Ethical Considerations

This study was approved by the CEU Cardenal Herrera University Ethics Committee (CEI18/115) and followed the fundamental principles established in the Declaration of Helsinki. Completion of the survey was anonymous and voluntary. All the participants were informed about the characteristics of the study in a participant information sheet provided in the link to the study before starting to complete the survey. After receiving this information, the participants gave their consent to participation by checking a specific box.

### Statistical Analysis

There are no prospective data on fertility-specific aspects of quality of life (QoL) of female patients with diagnosis of infertility during confinement. Therefore, the following assumptions were made to test the hypothesis that the fertility-specific aspects of QoL would be reduced as a result of confinement; in other words, the FertiQol-Total scores would be significantly decreased during this time. We performed a power analysis using the G^*^Power (v3.1.9.2, Heinrich-Heine-Universität Düsseldorf, Germany) program and found that 85 participants would provide 80% statistical power at a 5% significance level (for two-sided tests) for a medium effect size (*d* = 0.3).

Compliance with the assumption of normality was checked for each dependent variable using Shapiro–Wilks tests. These tests indicated that the data was not normally distributed in any of the variables we studied; therefore, the non-parametric Friedman test was used to examine the within-group differences among the 3 experimental conditions, including the pre-, during-, and post-confinement conditions, setting the significance level at *P* < 0.05. The Wilcoxon signed-rank test with Bonferroni correction was performed as a *post-hoc* test if the variance analysis test result was significant, and for the pairwise comparisons, a *P*-value < 0.017 (0.05/3) indicated statistical significance. SPSS version 18.0 for Windows (IBM Corp., Armonk, NY) was used for all the analyses.

## Results

The web-based survey was concluded on 28 October 2020; the questionnaire was sent to 124 infertile women and a total of 88 (70.9%) completed it. After validation of the data, 85 respondents were finally included in the study and 3 questionnaires were excluded because the participants responded to the survey after the study period had finished. The general characteristics (mean ± standard deviation) of the participants are shown in Table 1.

**Table 1:** Participants' general characteristics.

Age (years)	33.5 ± 3.7
Weight (kg)	66.0 ± 15.1
Height (cm)	163.1 ± 6.2
Body mass index (kg/m^2^)	24.7 ± 5.0
Time pursuing pregnancy	3.0 ± 2.7
**Educational level *n* (%)**	
Low	6 (7.1)
Intermediate	37 (43.5)
High	42 (49.4)
**Work during the confinement *n* (%)**	
Active work outside	33 (38.8)
Remote working	20 (23.5)
Cessation of activity	26 (30.6)
Unemployed	6 (7.1)

*Values are expressed as mean ± standard deviation and as number and percentage [n (%)] for educational level and work during the confinement*.

Values were expressed as the mean ± standard deviation and as a number and per-centage [n (%)] for educational level, work during the confinement, and smoking habits. Based on the Friedman test, there were significant differences (*P* < 0.05) between the three measurements (pre-, during, and post-confinement) for all the variables. Wilcoxon signed-rank test pairwise comparisons showed significant differences in FertiQol-Total, HADS-Anxiety, HADS-Depression, HADS-Total, MEDAS, vigorous activity, moderate activity, and walking activity variables (minutes per week), number of minutes spent sitting per day, and number of cigarettes smoked (per day; *P* < 0.017; Table 2).

**Table 2:** Results of the Wilcoxon signed-rank tests.

				**Pre vs. during**		**Pre vs. post**		**During vs. post**	
**Variables**	**Pre-confinement**	**During-confinement**	**Post-confinement**	***P***	***Z***	***P***	***Z***	***P***	***Z***
Means	8 (2)	8 (3)	8 (3)	0.416	−0.81	<0.001^*^	−3.70	0.009^*^	−2.62
Vigorous activity (min per week)	25 (140)	30 (300)	35 (165)	0.004^*^	−2.85	0.188	−1.32	0.006^*^	−2.74
Moderate activity (min per week)	50 (120)	30 (240)	40 (180)	0.004^*^	−2.87	0.028	−2.20	0.687	−0.40
Walking activity (min per week)	180 (330)	30 (300)	300 (330)	<0.001^*^	−3.84	0.088	−1.71	<0.001^*^	−4.23
Number of minutes spent sitting (per day)	300 (300)	480 (300)	300 (240)	<0.001^*^	−5.13	0.761	−0.30	<0.001^*^	−5.02
Cigarettes (per day)	0 (3)	0 (6)	0 (4)	0.027	−2.21	0.414	−0.82	0.001^*^	−3.47
HADS-Anxiety	8 (5)	9 (7)	8 (7)	<0.001^*^	−3.65	0.001^*^	−3.30	0.07	−1.81
HADS-Depression	3 (5)	5 (6)	4 (6)	<0.001^*^	−5.58	<0.001^*^	−4.41	0.007^*^	−2.69
HADS-Total	10 (10)	14 (13)	12 (13)	<0.001^*^	−4.92	<0.001^*^	−4.26	0.016^*^	−2.41
FertiQol-Total	69 (20.5)	69 (23)	69 (23.5)	0.046	−1.99	0.008^*^	−2.63	0.289	−1.06

*Values are expressed as medians and interquartile ranges in parenthesis (*

* *P < 0.017)*.

The FertiQol-Total score showed a decreasing trend during confinement and significantly decreased during the post-confinement period compared to pre-confinement. The HADS-Total showed significant differences between the three time points, with the worst values being reported during the confinement, followed by the post-confinement period. Interestingly, the MEDAS questionnaire showed that the participants reported better eating habits post-confinement compared to pre-confinement or during the confinement. Regarding physical activity habits, our results showed that during the confinement, the participants had engaged in significantly higher levels of vigorous and moderate activity and significantly lower levels of walking activity compared to the period pre-confinement. No significant differences in terms of physical activity habits were reported between the pre- and post-confinement time points.

Finally, the participants reported increased consumption of tobacco during the confinement compared to their pre- and post-confinement habits. A total of 37.6% of all the participants were smokers. Of these, 62.5% had increased their cigarette consumption during confinement, 20.6% had decreased it, and 14.7% had not changed their consumption compared to time pre-confinement. Women who increased their tobacco use smoked an average of 4.9 ± 2.8 more cigarettes during the confinement compared to their previous situation. Those who used less tobacco consumed an average of 4.2 ± 2.9 fewer cigarettes.

Regarding exercise, 55.2% of the participants indicated having engaged in physical exercise during the confinement. The remaining participants indicated that they had en-countered an obstacle to engaging in exercise: 27% because of an inadequate emotional state, 9.3% because of fatigue or laziness, 4.7% because of a lack of space, and 3.5% had not considered exercise a priority. Finally, Table 3 shows participant adherence to the Mediterranean diet (low, medium, or high adherence) and positive answers to MEDAS questionnaire, while Table 4 shows the results of the anxiety and depression subscales of the HADS questionnaire according to the severity of the reported symptoms.

**Table 3:** Adherence to the mediterranean diet and positive answers to the *Mediterranean Diet Adherence Screener* (MEDAS) questionnaire.

**Adherence to the** **Mediterranean diet**	**Pre-** **confinement**	**During** **confinement**	**Post-** **confinement**
LOW	15 (17.6)	12 (14.1)	5 (5.9)
MEDIUM	51 (60.0)	56 (65.9)	53 (62.3)
HIGH	19 (22.4)	17 (20)	27 (31.7)
Olive oil, main dressing	72 (84.7)	75 (88.2)	75 (88.2)
Olive oil, ≥ 4 tablespoons/day	54 (63.5)	56 (65.9)	55 (64.7)
Vegetables, ≥ 2 servings/day	32 (37.6)	41 (48.2)	43 (50.6)
Fruits, ≥ 3 servings/day	18 (21.2)	20 (23.5)	22 (25.9)
Read meat, <1 serving/day	62 (72.9)	61 (71.8)	66 (77.6)
Butter, <1 serving/day	80 (94.1)	80 (94.1)	82 (96.5)
Sweet beverages, <1 serving/day	70 (82.4)	66 (77.6)	72 (84.7)
Wine, 7 servings/week	3 (3.5)	10 (11.8)	3 (3.5)
Legumes, ≥ 3 servings/week	26 (30.6)	27 (31.8)	27 (31.8)
Fish and seafood, ≥ 3 servings/week	22 (25.9)	22 (25.9)	24 (28.2)
Sweets, <3 servings/week	47 (55.3)	44 (51.8)	51 (60.0)
Nuts, ≥ 3 servings/week	51 (60.0)	48 (56.5)	51 (60.0)
White meat preferred over red meat	72 (84.7)	67 (78.8)	73 (85.9)
Soffritto	54 (63.5)	54 (63.5)	53 (62.4)

*Data are expressed as number and a percentage in parenthesis [n (%)]*.

**Table 4:** The anxiety and depression categorisation levels based on the severity of the reported symptoms.

**HADS-Anxiety**	**Pre-confinement**	**During** **confinement**	**Post-confinement**
No anxiety	43 (50.6)	32 (37.6)	34 (40)
Mild	25 (29.4)	21 (24.7)	24 (28.2)
Moderate	12 (14.1)	22 (25.9)	18 (21.2)
Severe	5 (5.8)	10 (11.8)	9 (10.6)
**HADS-Depression**			
No depression	77 (90.6)	61 (71.8)	69 (80)
Mild	8 (9.4)	11 (12.9)	11 (12.9)
Moderate	0 (0)	11 (12.9)	5 (5.8)
Severe	0 (0)	2 (2.4)	0 (0)

*Data are expressed as number and a percentage in parenthesis (n (%)]*.

## Discussion

To the best of our knowledge, this is the first study to analyse the lifestyle and emotional well-being of infertile Spanish women during the COVID-19 pandemic. The data were collected through an online survey which was distributed between 1 September 2020 (the day the Reproduction Service in the hospital was reopened) and 28 October 2020 (when the state of alarm was again declared in Spain and new restrictions on mobility were put into place). To understand the effects of the confinement and impact of the closure and subsequent reopening of the reproduction services, we collected information about three moments during the pandemic so far: before confinement, during confinement, and when the reproductive care unit reopened. The response rate to the survey was 70, 9%. This result was comparable to previous response rates reported on this topic ranging between 29 and 86% (31–34). Our survey was anonymous, so it was not possible to follow up on those who did not respond. Besides, no financial incentive was provided to incentivize participation in this study.

The pre-conception period is key to identifying possible modifiable risk factors related to lifestyle that can make conception difficult. Among these factors, diet plays a fundamental role and can positively or negatively influence both reproductive health and future maternal-foetal health (35). Recent data suggest that good adherence to the Mediterranean diet, based on the consumption of large amounts of fruits, vegetables, legumes, and fish, and low intake of red meat and processed foods, increases the chances of success of reproductive treatments in terms of clinical pregnancy and live births (10). In this study, despite the foreseeable changes in diet as a consequence of confinement, we did not observe significant changes in adherence to the Mediterranean diet among these women compared to the pre-confinement time period. In line with our results, other authors also did not observe changes in the eating patterns of other population groups as a result of the confinement, as was observed in the case of pregnant women (36) and female desk workers (37).

Most of the participants showed medium level of adherence to the Mediterranean diet (65.9%) during confinement, while the remaining participants showed low (14.1%) or high (20%) adherence. It is worth noting that the Spanish Society for Community Nutrition (38) considers high adherence to the Mediterranean diet to be ideal. In this sense, only 20% of the participants complied with these recommendations compared to 80% who re-ported sub-optimal adherence levels. These results differed from those obtained in another study carried out in 140 Italian women planning to undergo assisted reproduction treatment, in which a worsening of the quality of their diets was observed (greater consumption of red meat, sweets, pastries, and sugary drinks) during confinement with respect to the previous situation (31). However, it should also be noted that the authors did not use a validated questionnaire, but rather, collected information on eating habits based on the recommendations of the Italian Society of Human Nutrition, while in this current study we used the MEDAS questionnaire which has been validated for the Spanish population. Thus, it is difficult to compare these studies because of the discrepancy in the instruments used to measure eating habits.

Interestingly, we observed a significant improvement in adherence to the Mediterranean diet when the reproduction service was reopened compared to pre-confinement and during the confinement. More participants reported high adherence (20% during confinement vs. 31.7% when the reproduction consultation was reopened) and fewer women re-ported low adherence (with a decrease from 14.1 to 5.9%, respectively). These favourable results in terms of diet after confinement could be related to the lower global levels of anxiety and depression (HADS-Total) reported by the participants when the reproduction service was reopened compared to during confinement. In fact, some authors have observed that infertile women with higher anxiety levels exhibited poorer eating behaviours (39). However, despite this observed improvement in diet, 68.2% of the participants showed adherence levels lower than recommended. Of note, an inappropriate dietary pattern can lead patients to become overweight or obese, which in turn is related to ovulatory disorders (40, 41) and a decrease in the success of fertility treatments (14, 15). In our study, 32.5% of the participants had a BMI above normal values. The data we obtained regarding the post-confinement diet indicated that, although the participants improved their adherence to the Mediterranean diet, the eating behaviour was sub-optimal in a high percentage of these patients.

Exercising is another of the recommendations established by the clinical guidelines for fertility during the preconception period (20, 21). In this study, more than half of the participants (55.2%) indicated that they had exercised during the confinement period. This result is similar to that obtained by Cirillo et al. who observed that of the 140 women planning to start reproductive treatment before the pandemic that they surveyed, 58% had continued to exercise during the confinement period (31). In our research, the remaining women indicated having encountered some form of obstacle to engagement in exercise, with the main obstacles they had perceived being an inadequate emotional state (27%), fatigue or laziness (9.3%), or lack of space (4.7%).

Regarding the amount of exercise performed, the participants had increased the time they spent engaging in vigorous exercise by 40% and in moderate exercise by 30% during confinement compared to the pre- and post-confinement situations. This increase placed the participants' level of exercise within those of the WHO recommendations on physical activity for health (42). These recommendations suggest performing 150–300 min of moderate activity or 75–150 min of vigorous activity, or a combination of the two per week, preferably distributed throughout the week. Our results showed that the participants performed an average of 24.4 min/day of vigorous exercise and 21.3 min/day of moderate exercise compared to the mean 15 min/day they had dedicated to each type of exercise before and after the confinement period. However, this increase in physical activity was accompanied by a significant decrease in the time spent walking, which went from an average of 342 min per week to 195 min during confinement, thereby representing a 43% reduction. In addition, during confinement an average of 7.2 h were spent engaged in sedentary activities compared to 5.4 h pre- and post-confinement. Consistent with our results, Cirillo et al. (31) obtained similar findings when they observed that more participants had been active during confinement (42.9%) compared to prior to confinement (30%), alongside an increase in sedentary behaviour in 40% of the women surveyed.

The beneficial effects of exercise on health were put in the spotlight during the pandemic, especially in countries where strict confinements were imposed, such as Spain. Given the detrimental effects of a sedentary lifestyle and physical inactivity on health, experts encouraged the population to exercise at home. The recommendations received by the population during the confinement, together with the fact that most of the participants had more time available to them during this time, may justify the participants' increased engagement in exercise. In another study carried out in a New Zealand population (in which an increase in physical activity was also observed during confinement), the participants indicated that one of the main reasons for this increase was that they had more time available to them because they no longer had to travel to their place of work (43). In our study, around 60% of the participants were in a similar situation in which, for various reasons (e.g., remote working, cessation of work activity, or unemployment) their outings were restricted to ‘essential activities' such as the purchase of essential products, foods, or medications. However, this increase in exercise was not maintained after confinement but rather, the participants returned to their previous levels in the post-confinement period.

On the other hand, despite the increase in the levels of vigorous and moderate exercise observed during the confinement, the participants showed higher scores in the HADS questionnaire. It must be taken into account that the confinement forced the exercise to be carried out indoors. In this sense, scientific evidence suggests that exercising outdoors, especially in green spaces, can favorably influence well-being compared to exercising indoors (44, 45). This fact, together with the overall decrease in the physical activity levels of the participants (greater sedentary lifestyle and less time walking) could partly explain the higher levels of anxiety and depression observed during the confinement.

Tobacco use is another lifestyle factor that negatively impacts female fertility. Tobacco consumption is related not only to an increased risk of cardiovascular disease, but also to infertility, low success rates in reproductive treatments, and a higher risk of miscarriage (46–49). Given these harmful effects on reproductive parameters, the guidelines recommend the cessation of this habit in smokers seeking pregnancy (20, 21). Our results showed a tendency towards increased tobacco consumption during confinement. This trend was confirmed when we carried out a sub-analysis of the data from female smokers. Specifically, we observed that 37.6% had a smoking habit and of these, 62.5% had in-creased their consumption during confinement. There were more smokers in our study than the National Health Survey data average, with 24.3% of them being in the 25–34 years age group (50), similar to the data for female tobacco users from a recent study on infertile women (26.6%) (51).

Moreover, women seem to be more susceptible to anxiety disorders under normal conditions and tend to smoke more than men to cope with stress (52). In the current context, Klemperer et al. used a survey on the consumption of tobacco and electronic cigarettes to analyse 366 participants and observed that 30% of them had increased the amount and frequency of consumption in response to the stress generated by the pandemic (53). In our study, together with the increase in tobacco consumption, we also observed higher levels of anxiety in confinement, which would justify our results regarding tobacco consumption. It is also worth mentioning that during the confinement caused by COVID-19, the Spanish authorities considered tobacco as an essential product and so tobacconists continued to open and citizens were allowed to leave their homes to purchase tobacco. Per-haps this fact also contributed to the increase in tobacco consumption among female smokers. When the reproduction unit was reopened, the participants reported having re-turned to their pre-confinement levels of consumption and 9.3% of these women had given up the habit. The abandonment of smoking by these participants may have been motivated by the temporal proximity of a possible fertility treatment.

In contrast, the participants showed significantly higher general levels of anxiety and depression during confinement, with a mean total score located at the cut-off point between moderate and severe levels (14.1 ± 4.9 out of 21 on the HADS scale). More than 60% had experienced anxiety symptoms during confinement (compared to 50.6% before confinement) with a higher proportion reporting medium and moderate levels. Furthermore, the symptoms of depression markedly increased during confinement (28.2 vs. 9.4% before the pandemic). Consistent with our results, other authors also observed an increase in the levels of anxiety and depression reported by infertile women from other countries during confinement. Indeed, Barra et al. observed symptoms of anxiety and depression in 45.5% of women in a survey of 524 infertile patients; more participants also reported moderate symptoms, with 1% describing severe symptoms (54). In another study conducted in 627 Italian infertile couples who were candidates for assisted reproduction treatments, confinement had produced a moderate/severe psychological impact, with these women reporting higher levels of anxiety and depression (33). Studies carried out during the pandemic on infertile women from other countries, such as the US (55, 56), Turkey (57), the United Kingdom (58), or Canada (59) also showed results consistent with an increase in anxiety and depression. When the reproductive service was reopened, we observed a significant decrease in the overall HADS questionnaire score. However, the anxiety subscale results showed that the participants had maintained levels of anxiety similar to those shown during confinement.

Regarding quality of life related to fertility, there was a clear downward trend during confinement which became even more evident when the reproductive services were reopened. We must consider that our study included women who had been referred to the hospital's reproduction service because they were having difficulty in achieving pregnancy naturally, and so the average age of the participants was lower than that of the women who usually undergo *in vitro* fertilisation treatments (60). In this sense, the results of research analysing the predictors of quality of life in infertile couples showed an association between advanced age and lower scores on the FertiQol scale (61). Likewise, infertility over longer periods and low educational levels were two other variables that were also associated with a lower quality of life among infertile women (61–63). The women included in our study had been trying to become pregnant for a shorter period of time compared to the mean of all the patients undergoing IVF (60), and only 7.1% of the participants had a low level of education. Thus, perhaps these data could justify the fact that the decrease in quality of life observed in our study participants during the COVID-19 pandemic was not as marked as we had expected during the confinement period.

The greater anxiety and lower quality of life observed at the time of reproductive service reopening may have been related to the length of the unit's closure. The overburden experienced by the hospital as a result of the high number of patients with COVID-19 had forced the hospital to keep the reproduction service closed for a total of 6 months. Indeed, other recent studies have also described the discomfort experienced by a large percentage of couples when fertility treatments were suspended because of the pandemic (23, 32, 58) as well as the desire of almost 50% of these patients to immediately restart treatment (32). Notwithstanding, scientific evidence suggests that stress can negatively impact female fertility, and its presence is associated with lower conception rates, long menstrual cycles (> 35 days), and poorer fertility treatment results (64–67). In this sense, the negative emotional responses observed as a consequence of the pandemic would represent an added risk factor for the reproductive health of the participants.

Our results provide novel information on the impact that the pandemic and the closure of reproductive services had on the lifestyle and emotional well-being of infertile women. In addition, the data show that despite the recommendations established by the fertility clinical guidelines on pre-conception care, our participants had generally demonstrated un-healthy habits even before confinement, especially with regard to diet and tobacco consumption. In light of the results obtained in this work, the implementation of specific online programs to promote healthy lifestyles and to help improve the reproductive parameters of women with fertility problems would seem to be prudent. Online interventions have the advantage of being just as effective as traditional ones (68, 69) and of having a good cost-benefit ratio (70). Furthermore, these types of interventions can be self-administered by patients themselves and so would not imply their suspension if there were another Reproduction Unit shut down, or a similar event. Future randomised clinical trial research should expand the analysis of the effects of online interventions focused on promoting healthy lifestyles for women with fertility problems.

This study had some limitations such as recall bias because all the participants' responses were conditioned by their ability to recall their habits, as well as desirability bias, whereby participants tended to minimise unhealthy habits and exaggerate healthy behaviours. On the other hand, it should be borne in mind that the HADS questionnaire collects information on levels of anxiety and depression but does not allow us to differentiate between trait anxiety and state anxiety. In addition, the total number of participants included in this study was not remarkably high. However, we must consider that this survey was disseminated to the participants when they were given new appointments at the hospital after the reproductive service had reopened, and at that time, to guarantee safety and the distance between patients, the number of appointments per day was considerably reduced. In addition, the declaration of a new state of alarm with new mobility restrictions on 28 October 2020, may have altered the results of our study by forcing us to end the data collection period before its planned endpoint.

## Conclusions

In conclusion, confinement increased the levels of anxiety and depression reported by infertile women and produced an increase in tobacco use among smokers in this population. In addition, during confinement the women had spent more time sitting and spent less time walking. However, this decline was accompanied by an increase in levels of vigorous and moderate physical activity. The reopening of the Reproduction Unit did not reduce the anxiety levels of the participants, but these women did decrease their tobacco consumption and improved their adherence to the Mediterranean diet, which had not been altered by confinement compared to the situation prior to the pandemic. The quality of life of this population group was diminished after the prolonged period of re-productive care closure.

## Data Availability Statement

The original contributions presented in the study are included in the article, further inquiries can be directed to the corresponding authors.

## Ethics Statement

The studies involving human participants were reviewed and approved by this study was approved by the CEU Cardenal Herrera University Ethics Committee (CEI18/115). The patients/participants provided their written informed consent to participate in this study.

## Author Contributions

GB-R and JL conceived this research methodology and wrote/prepared the original draft. AB-R and PM-B were responsible for the methodology. RB-S and LS-R conducted a formal analysis. ED-D managed the investigation. RB-S and AB-R reviewed and edited the manuscript. PM-B, LS-R, and ED-D were responsible for visualization. GB-R acquired funding. All authors contributed to the article and approved the submitted version.

## Conflict of Interest

The authors declare that the research was conducted in the absence of any commercial or financial relationships that could be construed as a potential conflict of interest.
